# Synthesis of naturally-derived macromolecules through simplified electrochemically mediated ATRP

**DOI:** 10.3762/bjoc.13.243

**Published:** 2017-11-20

**Authors:** Paweł Chmielarz, Tomasz Pacześniak, Katarzyna Rydel-Ciszek, Izabela Zaborniak, Paulina Biedka, Andrzej Sobkowiak

**Affiliations:** 1 Department of Physical Chemistry, Faculty of Chemistry, Rzeszów University of Technology, Al. Powstańców Warszawy 6, 35-959 Rzeszów, Poland

**Keywords:** flavonoids, on-demand *se*ATRP, quercetin-based macromolecules

## Abstract

The flavonoid-based macroinitiator was received for the first time by the transesterification reaction of quercetin with 2-bromoisobutyryl bromide. In accordance with the “grafting from” strategy, a naturally-occurring star-like polymer with a polar 3,3',4',5,6-pentahydroxyflavone core and hydrophobic poly(*tert*-butyl acrylate) (P*t*BA) side arms was synthesized via a simplified electrochemically mediated ATRP (*se*ATRP), utilizing only 78 ppm by weight (wt) of a catalytic Cu^II^ complex. To demonstrate the possibility of temporal control, *se*ATRP was carried out utilizing a multiple-step potential electrolysis. The rate of the polymerizations was well-controlled by applying optimal potential values during preparative electrolysis to prevent the possibility of intermolecular coupling of the growing polymer arms. This appears to be the first report using on-demand *se*ATRP for the synthesis of QC-(P*t*BA-Br)_5_ *pseudo*-star polymers. The naturally-derived macromolecules showed narrow MWDs (*Đ* = 1.08–1.11). ^1^H NMR spectral results confirm the formation of quercetin-based polymers. These new flavonoid-based polymer materials may find applications as antifouling coatings and drug delivery systems.

## Introduction

In the last decade, there have been increasing research activities in the use of atom transfer radical polymerization (ATRP) to prepare naturally-derived star-like polymers [1–4]. Considering this method, naturally-occurring polymers can be synthesized via three main strategies: “grafting onto” [5–10], “grafting through” [11–12], or “grafting from” [10,13–21]. The “grafting from” approach in particular, allows the tailoring of the side chain composition and the introduction of functional groups via polymerization [21]. This technique consists in the application of a multifunctional macromolecule. The number of initiating groups on this macromolecule codes the number of arms in the synthesized star polymer. Moreover, by extension of ω-chain ends on the periphery of the star we can easily introduce a next segment to the polymer [22–23].

This article aims at the synthesis of quercetin-based star-like polymers with a polar quercetin (QC) core and hydrophobic poly(*tert*-butyl acrylate) (P*t*BA) arms which has not yet been reported. Quercetin with five terminal hydroxy groups was chosen as an efficacious solution to receive functionalised polymers. It is a naturally occurring flavonoid, which is abundantly found in citrus fruits, herbs, vegetables, seeds, tea, nuts, and red wine [24–26]. It is considered to be a strong antioxidant due to its ability to scavenge free radicals and bind transition metal ions [27]. Quercetin inhibits xanthine oxidase [27–29], inhibits lipid peroxidation in vitro [27–28,30], and scavenges oxygen radicals [27–28,31–33]. There is a tremendous importance of this antioxidant in the prevention of a range of cardiovascular diseases [27,34–35], cancer [26–27,36], and neurodegenerative diseases [27]. P*t*BA was selected as functional arm of polymer stars because it can be readily transformed to poly(acrylic acid) via deprotection, yielding polyelectrolytes. Such polymers are one of the most extensively studied, industrially important, water-soluble macromolecules [37–39], widely used as dental adhesives, controlled release devices, coatings, and in pharmaceutical industry [40–41]. Therefore, it is expected that these synthesized naturally-derived macromolecules can become key elements of antifouling coatings and drug delivery systems.

ATRP is one of the most versatile techniques that allow obtaining a wide range of polymers with controlled composition, molecular weight (MW), molecular weight distribution (*M*_w_/*M*_n_, MWD, *Ð*), and degrees of polymerization (DP) [42–53]. Significant efforts have been dedicated to the development of the “green chemistry” variety of this method. The catalyst complex concentration has been substantially reduced to parts per million (ppm) level in the reaction system, due to the development of the simplified electrochemically mediated ATRP (*se*ATRP) approach [54], which offers elimination of chemical reducing agents, catalyst recycle possibility, and an option to receive polymers with narrow MWD [55–56]. Additionally, application of external stimuli offered a possibility of temporal control, such as the stopping and restarting of the polymerization by switching the “off” and “on” stages, respectively [53,55], while maintaining the well-controlled characteristic of the process [55–57]. A similar effect was received by turning the light source “on” and “off” in the photoATRP approach [58]. However, in this case, substantial light scattering could interfere or even prevent efficient polymerization [56]. Therefore, *se*ATRP offers a new opportunity to synthesize well-defined star-like polymers with predefined molecular structure.

The main objective of this study is to present the first example of a synthesis of a macromolecule initiator from the group of flavonoids and with well-defined star-like polymers, consisting of a quercetin core and hydrophobic P*t*BA arms with narrow MWDs by ATRP under multiple-step potential electrolysis conditions.

## Results and Discussion

**Figure 1:**
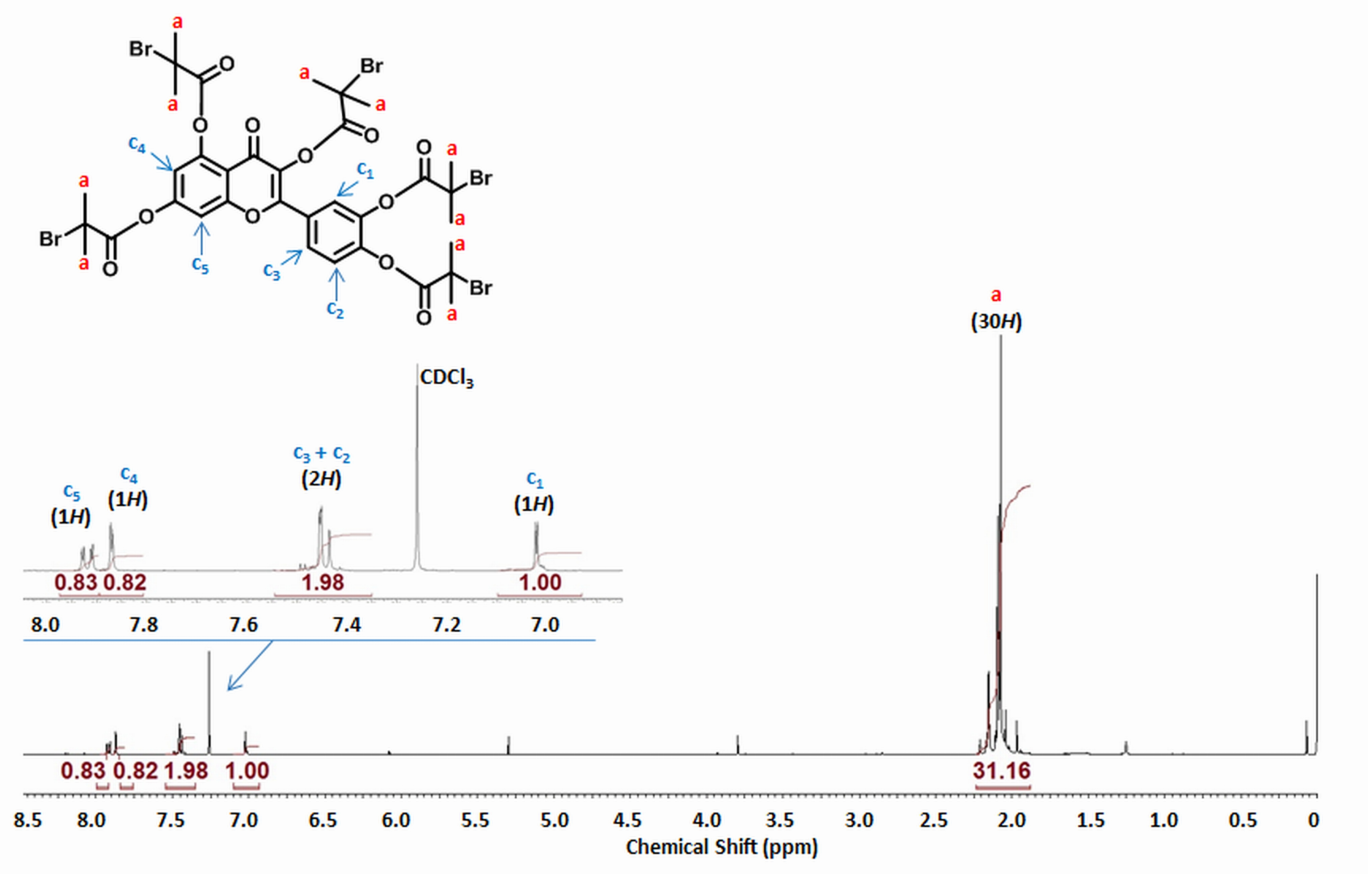
^1^H NMR analysis of QC-Br_5_ (*M*_n_ = 1,050, *Ð* = 1.11) after purification (in CDCl_3_).

A flavonoid-based macromolecule initiator with 5 Br atoms (QC-Br_5_) was synthesized by the transesterification reaction (Figure S1, Supporting Information File 1; *M*_n_ = 1,050, *M*_w_/*M*_n_ = 1.02). The chemical structure of QC-Br_5_ was confirmed by ^1^H NMR (Figure 1): δ (ppm) = 1.88–2.24 (30*H*, C*H*_3_–, a_1_), 6.93–7.10 (1*H*, =C*H*–, c_1_), 7.35–7.54 (2*H*, =C*H*–, c_2_ and c_3_), 7.81–7.90 (1*H*, =C*H*–, c_4_), and 7.91–7.98 ppm (1*H*, =C*H*–, c_5_). The degree of substitution of the hydroxy groups of 3,3',4',5,6-pentahydroxyflavone was determined by the area ratio of the methyl protons at the regions of δ = 1.88–2.24 ppm (30*H*) to the 1-benzene protons at the region of δ = 6.93–7.10 ppm (1*H*). According to this analysis, the quercetin-based product has 5 Br functionalities.

Cyclic voltammetry was used for the electrochemical characterization of the QC (Figure S2, Supporting Information File 1), QC-Br_5_ (Figure S3), and Cu^II^Br_2_/tris(2-pyridylmethyl)amine (TPMA) in the absence (Figures S5 and S6) and in the presence of QC-Br_5_ (Figures S7 and S8), all the reaction solutions contained *N*,*N*-dimethylformamide (DMF) and *tert*-butyl acrylate (*t*BA). One can observe, that quercetin is not reduced in the applied potential window (Figure S2, Supporting Information File 1), meanwhile it shows two small anodic peaks at 0.2 V and 0.5 V. According to the commonly accepted mechanism, both for water-containing [28,59] and for aprotic media [28,60–61] the first oxidation peak corresponds to the oxidation of the catechol moiety, the 3’,4’-dihydroxy group of quercetin, while the second peak relates to the oxidation of the –OH substituent next to the carbonyl group of quercetin. Brominated quercetin is electrochemically inactive between –0.75 V and 0.75 V (Figure S3, Supporting Information File 1). As expected, the Cu^II^Br_2_/TPMA catalyst complex is quasi-reversibly reduced to Cu^I^Br/TPMA at −0.3 V (Figure S5, Supporting Information File 1). The peak current for the cathodic peak (–0.3 V) and reverse anodic peak (–0.2 V) increases linearly with correlation coefficients 0.999 and 0.996 for the corresponding regression lines (Figure S6, Supporting Information File 1). The high linearity indicates that the processes are controlled by the rate of diffusion of the electroactive substance into the electrode surface. However, the separation of the peaks is characteristic for a quasi-reversible process at each scan rate applied. After introduction of QC-Br_5_ to the system containing Cu^II^Br_2_/TPMA complex a higher wave-shaped cathodic response was observed (Figure S7, Supporting Information File 1). Due to the fact that QC-Br_5_ is not reduced electrochemically at least to −0.75 (Figure S3, Supporting Information File 1), solely Cu^II^Br_2_/TPMA can be reduced at −0.3 V in electrochemical systems, effectuating subsequent, fast chemical reduction of QC-Br_5_ and regeneration of Cu^II^Br/TPMA. As expected, addition of an alkyl halide initiator to a solution of Cu^II^Br_2_/TPMA during the voltammetric measurements causes a loss of reversibility and an increase of the cathodic current because of reduction of the regenerated Cu^II^Br_2_/TPMA via the catalytic electrochemical catalytic process (EC’) (Figure S7, Supporting Information File 1) [55]. The disturbance from linearity for the dependence of the peak current on the square root of the scan rate (Figure S8, Supporting Information File 1) indicates the distinct non-diffusional component of the process, related to consecutive chemical regeneration of the Cu^II^Br_2_/TPMA complex.

To investigate the kinetics of the electrochemical catalytic process, the dependence of the ratio (catalytic current)/(reduction peak current in the absence of QC-Br_5_) – for the peak at −0.3 V, on the square root of different QC-Br_5_ concentration was analyzed (Figures S9 and S10, Supporting Information File 1). The dependence was linear (*R* = 0.997). The rate constant of the chemical reaction between the Cu^I^ complex and QC-Br_5_, i.e., the C’ reaction of the catalytic process (EC’), using the equations from the classic works of Savéant, Vianello [62] and Nicolson, Shine [63] was calculated. Dividing the equation for the peak of the catalytic current by the Randles-Sevčik equation for the quasi-reversible peak (for 298 K), we obtain


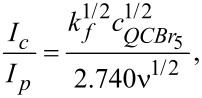


where *ν* is a scan rate. Because


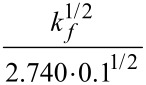


is the slope of the regression line for *I_c_/I_p_ = f(c_QC-Br5_)*^1/2^ dependence (equal 58.1), the calculated *k_f_* is 2.5·10^3^ M/s.

Flavonoid-based *pseudo*-star polymers with a quercetin core and P*t*BA side chains have been synthesized for the first time using only 78 ppm by weight (wt) of Cu^II^ complex, following the *se*ATRP procedure (Table 1).

**Table 1:** Summary of quercetin-based *pseudo*-star polymers synthesis by *se*ATRP.

entry	[M]/[MI]/ [Cu^II^Br_2_]/[TPMA]	*E*_app_^a^	*k*_p_^app^ (h^-1^)^b^	conv (%)^b^	DP_n,theo_ (arm)^b^	*M*_n,theo_ (×10^-3^)^c^	*M*_n,app_ (×10^-3^)^d^	*M*_w_/*M*_n_^d^

1	110/1/0.011/0.022	*−*240 mV	0.471	73	80	52.6	36.6	1.08
2	110/1/0.011/0.022	*Multi-constant E_app_ Electrolysis*^e^	0.452^f^	75	82	53.6	37.3	1.11

General reaction conditions: *T* = 65 °C; *V*_tot_ = 16 ml; *t* = 3 h [except entry 2: *t* = 6 h (“on” stages = 3 h; “off” stages = 3 h)]; [M]: [*t*BA] = 3.4 M; [MI]: [QC-Br_5_] = 6.2 mM calculated per 5 Br initiation sites; [Cu^II^Br_2_/TPMA] = 0.34 mM; [tetrabutylammonium perchlorate (TBAP)] = 0.2 M. Constant potential *se*ATRP: entry 1; Controlled multi-constant potential *se*ATRP: entry 2. ^a^Applied potentials (*E*_app_) were selected based on cyclic voltammetry (CV) analysis of catalytic complex (Figures S5 and S7, Supporting Information File 1); ^b^Monomer conversion, apparent propagation constants (*k*_p_^app^), and apparent theoretical degree of polymerization of monomer unit per arm (DP_n,theo_) were determined by NMR [64]; ^c^*M*_n,theo_ = ([M]_0_/[MI]_0_) × conversion × *M*_monomer_ + *M*_macroinitiator_; ^d^apparent *M*_n_ and MWD were determined by GPC; ^e^controlled potential program (*E*_app_ = *–*240 mV for the “on” stage and *E*_app_ = 600 mV for the “off” stage vs SCE); ^f^only for the “on” stages.

**Figure 2:**
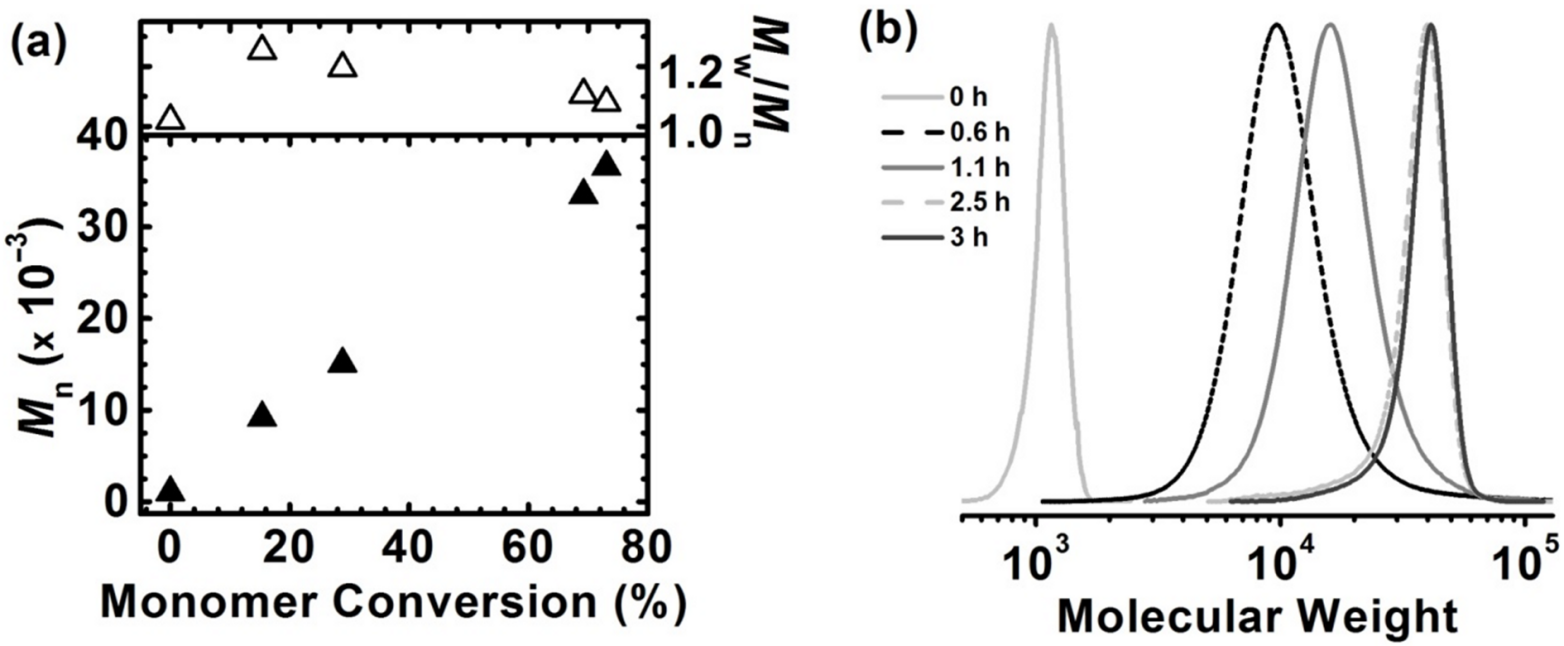
Synthesis of P*t*BA homopolymers grafted from quercetin-based macroinitiator via *se*ATRP under constant potential conditions; (a) *M*_n_ and *M*_w_/*M*_n_ vs monomer conversion, and (b) GPC traces of *t*BA polymerization and their evolution over time*.* Reaction conditions: [*t*BA]/[QC-Br_5_ (per 5 initiation sites)]/[Cu^II^Br_2_]/[TPMA] = 110/1/0.011/0.022, [*t*BA] = 3.4 M, [Cu^II^Br_2_/TPMA] = 0.34 mM, [TBAP] = 0.2 M, *T* = 65 °C. Table 1, entry 1.

The synthesis of quercetin-based macromolecule initiator with 5 side arms of P*t*BA under constant potential preparative electrolysis conditions was realized (Table 1, entry 1, Figure 2a,b and Figures S11a, and S11b, Supporting Information File 1). At the beginning of the constant potential polymerization approach (Figure S11a, Supporting Information File 1), merely deactivator is prevalent, thus the cathodic current decays as deactivator is transformed to an activator, after which it achieves constant value corresponding to the deactivator/activator ratio, adjusted by *E*_app_ [55]. The rate of the electrolysis was well-controlled by applying optimum *E*_app_ values in order to preclude coupling of side chains [64–65]. The first-order kinetic relationship (Figure S11b, Supporting Information File 1) was observed. Figure 2a,b shows that MW increased linearly with conversion and that narrow MWD course toward higher MW was achieved.

**Figure 3:**
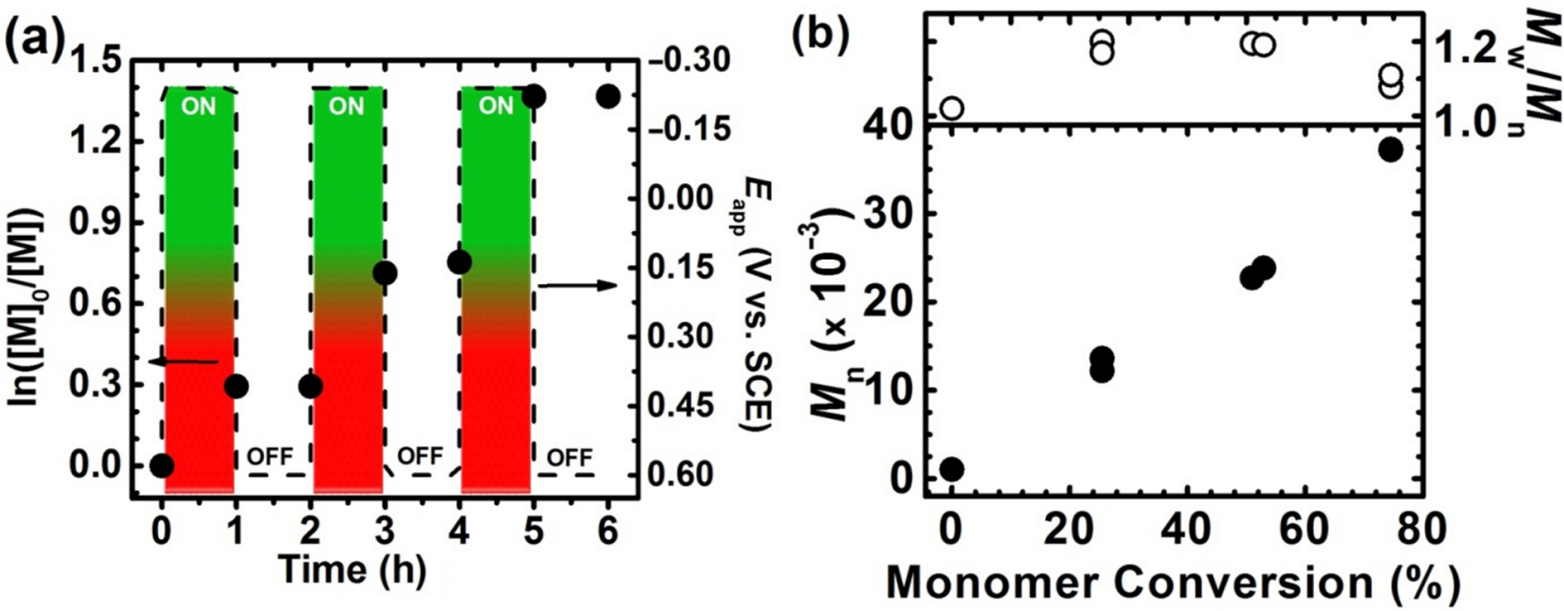
(a) First-order kinetic plot of *se*ATRP with periodically applied different values of potential, between −240 mV and 600 mV vs SCE, respectively; and (b) *M*_n_ and *M*_w_/*M*_n_ with respect to monomer conversion. Reaction conditions are identical to those stated in Figure 2.

To verify the living character of the electrochemically mediated process, the sufficient applied potential was imposed to repetitively switch the system between active and dormant states according to previous research [55,57]. This was achieved by cycling *E*_app_ between −0.29 V and 0.55 V vs SCE (Table 1, entry 2, Figure 3a,b, and Figures S12a and S12b, Supporting Information File 1). The first of these potentials favors formation of Cu^I^ at the electrode and hence polymerization was activated, whereas the second potential, being more positive than *E*_1/2_, promotes Cu^II^ regeneration and leads to a dormant species of propagating radicals (Figure S12a, Supporting Information File 1) [57]. This potential cycle was repeated three times, efficiently increasing the monomer conversion to 25, 51, and then to 75% during active periods (Figure 3a). MW steadily increased during the “on” periods, while no low-MW polymers were detected (Figure 3b and Figure S12b, Supporting Information File 1). These observations illustrate the living characteristics of the polymerization with regard to efficient reinitiation of chain ends, owing to the applicable preservation of chain-end functionality [57]. Such “pausing” of the reaction could be beneficial in preparation of wide range of naturally-derived macromolecular stars and brushes with predictable molecular weights of the polymer grafts.

The chemical structure of the received QC-(P*t*BA_82_-Br)_5_ *pseudo*-star-shaped polymer (Table 1, entry 2) was confirmed by ^1^H NMR spectroscopy (Figure S13, Supporting Information File 1). The chemical shifts, 1.10–2.00 ppm and 2.10–2.40 ppm, are ascribed to the –C*H*_2_– (β), –C*H*_3_ (b), and –C*H*– (α) groups of the P*t*BA units, denoting the inherence of P*t*BA arms [48,66–68].

## Conclusion

Naturally-derived macromolecules were synthesized based on a new strategy including the synthesis of a 3,3',4',5,6-pentahydroxyflavone-based core with 2-bromoisobutyryl bromide as initiation molecule, and grafting of the P*t*BA arms of the flavonoid-based moiety by facile *se*ATRP technique. To demonstrate the possibility of temporal control, on-demand *se*ATRP was carried out utilizing multiple-step potential electrolysis. The feasibility of the electrochemical switch exploitation for the control of cooper oxidation states and therefore activation or deactivation of the polymerization was demonstrated by the sequence of repeated stepping *E*_app_ from −0.24 V to 0.60 V vs SCE. Such “pausing” of the reaction could be beneficial in preparation of more complex architectures with predictable molecular weights of the polymer grafts. This is the first report announcing using simplified constant potential and multi-step constant potential mediated ATRP for the synthesis of flavonoid-based star-like polymers. The results of GPC, and ^1^H NMR prove the successful preparation of the star-shaped polymers. These new polymer materials create potential possibilities of using them as key elements of biologically active thin films in tissue engineering and as drug delivery systems.

## Supporting Information

File 1 Experimental section including NMR spectra, first-order kinetic plot, GPC traces, preparative electrolysis and CV results.
