# Supplementary material for: Synthesis of naturally-derived macromolecules through simplified electrochemically mediated ATRP
Source: Beilstein J Org Chem. 2017 Nov 20;13:2466–72. doi: 10.3762/bjoc.13.243 (PMC5704770; doi:10.3762/bjoc.13.243)
Supplement: File 1 — Experimental section including NMR spectra, first-order kinetic plot, GPC traces, preparative electrolysis and CV results. [file Beilstein_J_Org_Chem-13-2466-s001.pdf]

Supporting Information

for

Synthesis of naturally-derived macromolecules through simplified electrochemically mediated ATRP

Paweł Chmielarz*, Tomasz Pacześniak, Katarzyna Rydel-Ciszek, Izabela Zaborniak, Paulina Biedka and Andrzej Sobkowiak

Address: Department of Physical Chemistry, Faculty of Chemistry, Rzeszów University of Technology, Al. Powstańców Warszawy 6, 35-959 Rzeszów, Poland

Email: Paweł Chmielarz* - p_chmiel@prz.edu.pl

*Corresponding author

Experimental section including NMR spectra, first-order kinetic plot, GPC traces, preparative electrolysis and CV results

Experimental

Materials

Quercetin (QC, M_n = 302.24, ≥95%), 2-bromoisobutyryl bromide (BriBBr, 98%), *N*-methyl-2-pyrrolidone (NMP, >99%), dichloromethane (DCM, >99.9%), sodium hydrogencarbonate ($NaHCO_3$, >99.7%), magnesium sulfate ($MgSO_4$, >99.5%), tetrahydrofuran (THF, >99.9%), methanol (MeOH, >99.8%), tetrabutylammonium perchlorate (TBAP, >98%), copper(II) bromide ($Cu^{II}Br_2$, 99.9%) were purchased from Aldrich. *N*,*N*-Dimethylformamide (DMF, 99.9%) was purchased from Acros. These reagents were used without further purification. Tris(2-pyridylmethyl)amine (TPMA) and $Cu^{II}Br_2$/TPMA catalyst complex were prepared according to references [1,2]. *tert*-Butyl acrylate (*t*BA, >99% from Aldrich) was passed through a column filled with basic alumina prior to use to remove inhibitor. Platinum (Pt) wire, Pt mesh and Pt disk (3 mm diameter, Gamry) were purchased from Alfa Aesar (USA). Cyclic voltammetry (CV) and electrolysis were conducted in a Gamry cell kit (USA).

Analysis

^{1}H NMR spectra in $CDCl_3$ were measured in a Bruker Avance 500 MHz spectrometer. Monomer conversion and theoretical number-average molecular weight ($M_{n,th}$) were received from NMR analysis [3-5]. MWs and MWDs were acquired by Viscotek T60A GPC (guard, 10^5, 10^3, and 10^2 Å PSS columns; THF as eluent, flow rate 1.00 ml/min, PS calibration). Cyclic voltammetry (CV) was performed using Autolab model AUT84337 potentiostat running with a GPES software. The experiments were conducted in a two-compartment cell with a glass frit divider. The

working electrode (WE) was a platinum disk (0.008 cm^2), carefully polished with 0.05 µm alumina suspension (0.05 µm, Buehler) before every single measurement. The reference electrode (RE) was saturated calomel electrode (SCE), equipped with a saturated salt bridge and a Vycor tip, immersed inside a Luggin capillary, and placed together with a working electrode in the compartment containing the examined system. The counter electrode (CE) was a platinum plate (2 cm^2), located in the second compartment, containing solvent and supporting electrolyte. The Teflon cups were sealed with Teflon tape and dioxygen was removed with an argon-purge system. The electrolyses were recorded on a Metrohm Autolab potentiostat using a platinum mesh (A = ≈6 cm^2) as the working electrode and Al wire (l = 10 cm, d = 1 mm) as the counter electrode. During polymerizations a condenser was connected to the reaction cell kit and the temperature was maintained at 50 °C using a circulating thermostat (Labo Play ESM-3711-H). Values for potentials adapted for electrolysis were received from CV measurements at a 100 mV/s scan rate using SCE as RE.

Synthesis of the penta-functional 2-bromoisobutyrate-terminated quercetin ($QC\text{-}Br_5$) macroinitiator

The process of flavonoid-based macromolecule ATRP initiator synthesis was shown in Figure S1. Quercetin (2 g, 6.6 mmol) was dissolved in NMP (50 ml) under Ar atmosphere in a 100 ml round bottomed flask, stirred for 2 hr at room temperature and cooled to 0°C. A solution of BriBBr (28.6 ml, 231.6 mmol) in NMP (18 ml) was added dropwise over a period of 2.5 h, then the reaction solution was stirred for 8 days at room temperature. Upon completion, the product was diluted with DCM (30 ml) and washed with water (70 ml × 8), saturated $NaHCO_3$ aqueous solution (70 ml × 8), dried ($MgSO_4$), filtered and concentrated under reduced pressure. The resulting white solid product was dried under vacuum (5.68 g, yield 82%). The final

macromolecule ATRP initiator was characterized using ^1H NMR and CV (before – Figure S2 and after transesterification – Figure S3).

Synthesis of QC-(P*t*BA-Br)$_5$ *pseudo*-star polymers by *se*ATRP

The synthesis of QC-(P*t*BA-Br)$_5$ *pseudo*-star homopolymer was conducted via constant potential preparative electrolysis with stirring during the polymerization (Figure S4). The Pt mesh WE, Al wire CE, and SCE RE were prepared and located in the polymerization cell. 1.09 g of TBAP (3.2 mmol), 8 ml of *t*BA (55 mmol), 6.5 ml of DMF and 109 µL of $Cu^{II}Br_2$/TPMA solution (0.05 M in DMF) were introduced to the cell at 65 °C under a slow Ar purge. Then, 104 mg of QC-Br_5 (0.5 mmol) in 1.5 ml of DMF was added. Before the electrolysis the CV was recorded to determine the applied potential (E_{app} = −240 mV; Figures S5–S8) used subsequently during *se*ATRP procedure. The samples were withdrawn periodically to follow the monomer conversion, using ^1H NMR. The products were purified by dialysis against water and MeOH (MWCO 1000 Spectra/Por dialysis membrane), dried under vacuum for 1 day, dissolved in THF, passed through a neutral alumina column, and dried under vacuum for 11 days. The final product was characterized using GPC.

Temporal control in synthesis of QC-(P*t*BA-Br)$_5$ *pseudo*-star polymers by *se*ATRP

*se*ATRP with periodically applied different values of potential (E_{app} = –240 mV for the “on” stage and E_{app} = 600 mV for the “off” stage vs SCE) was applied. Reaction conditions were as follows: [*t*BA] = 3.4 M, [QC-Br_5] = 6.2 mM, [$Cu^{II}Br_2$/TPMA] = 0.34 mM, [TBAP] = 0.2 M, T = 65 °C, V_{tot} = 16 ml. An identical methodology as for the constant potential approach was used for the preparation of the reaction kit, the order of reagents introduction, the purification and for the monomer conversion, M_n, and

M_w/M_n determination. The final product was characterized using GPC and ^{1}H NMR (Figure S13) [6].

Additional experimental data

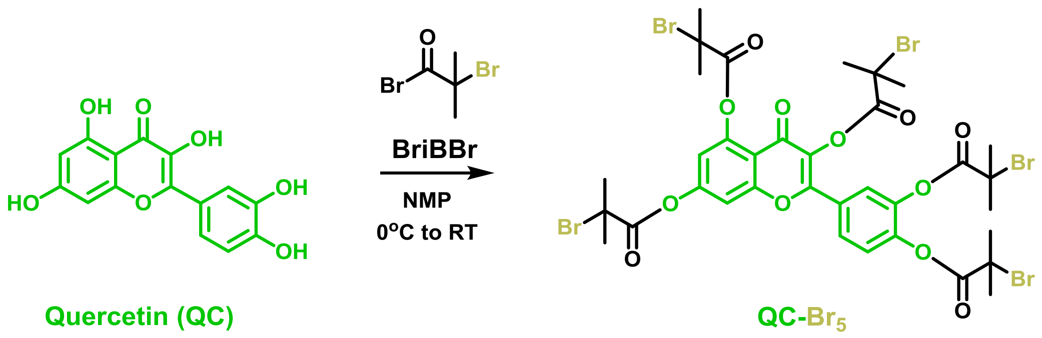

Figure S1: Synthesis of flavonoid-based QC-Br_5 macromolecule ATRP initiator.

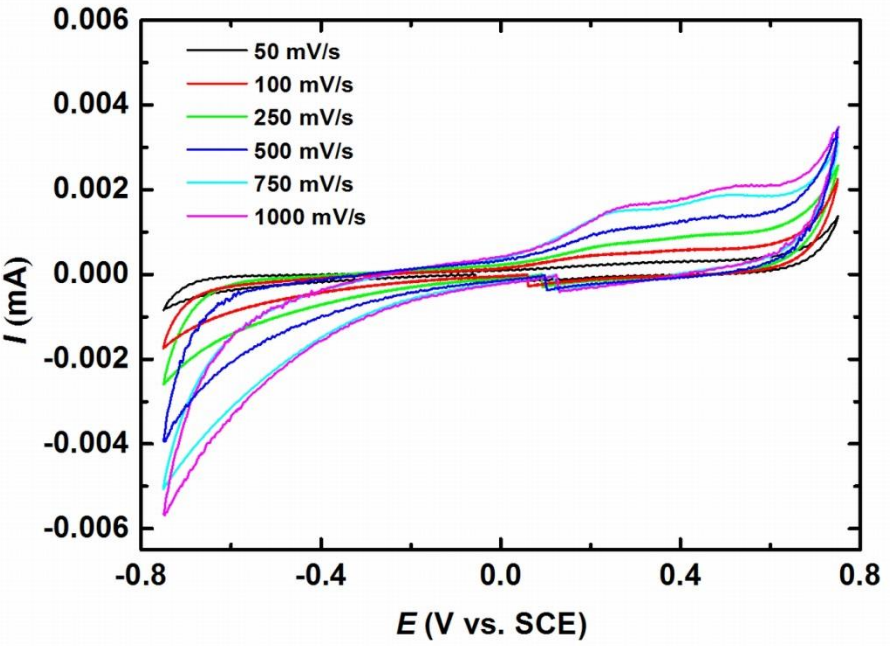

Figure S2: Cyclic voltammograms (cathodic scans) of 6.2 mM QC in 50% (v/v) *t*BA/DMF ([*t*BA] = 3.4 M) containing 0.2 M TBAP recorded at different scan rates (given next to the curves).

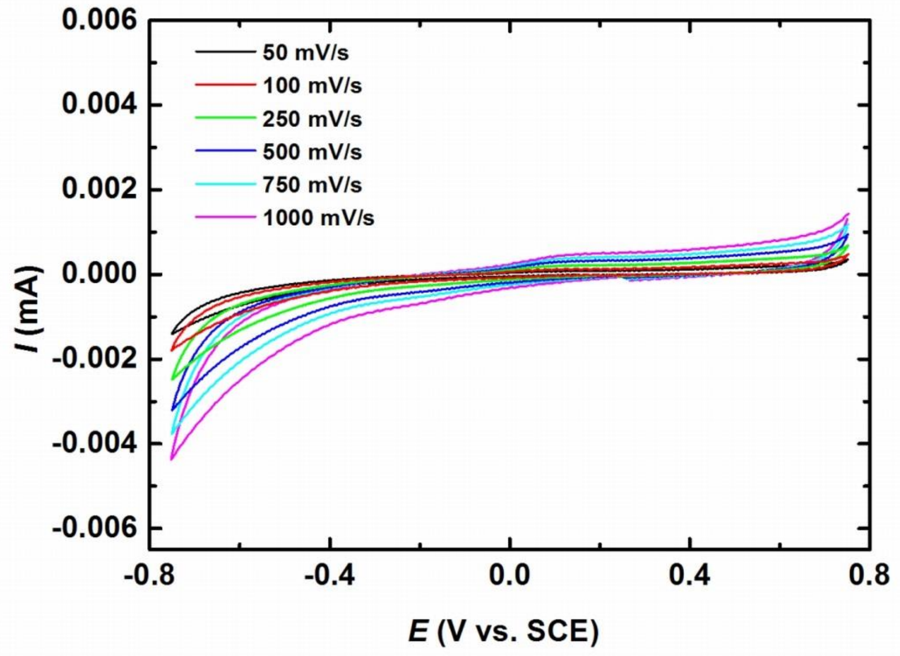

Figure S3: Cyclic voltammograms (cathodic scans) of 6.2 mM QC-Br_5 in 50% (v/v) *t*BA/DMF ([*t*BA] = 3.4 M) containing 0.2 M TBAP recorded at different scan rates (given next to the curves).

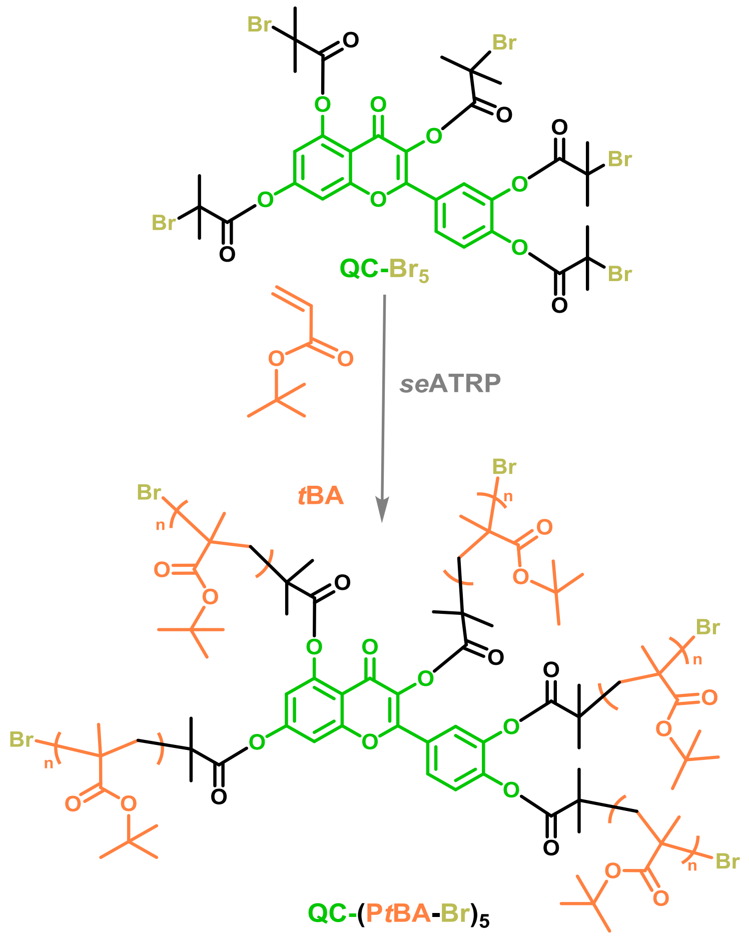

Figure S4: Synthesis of P*t*BA by grafting form QC-Br_5 macroinitiator via *se*ATRP.

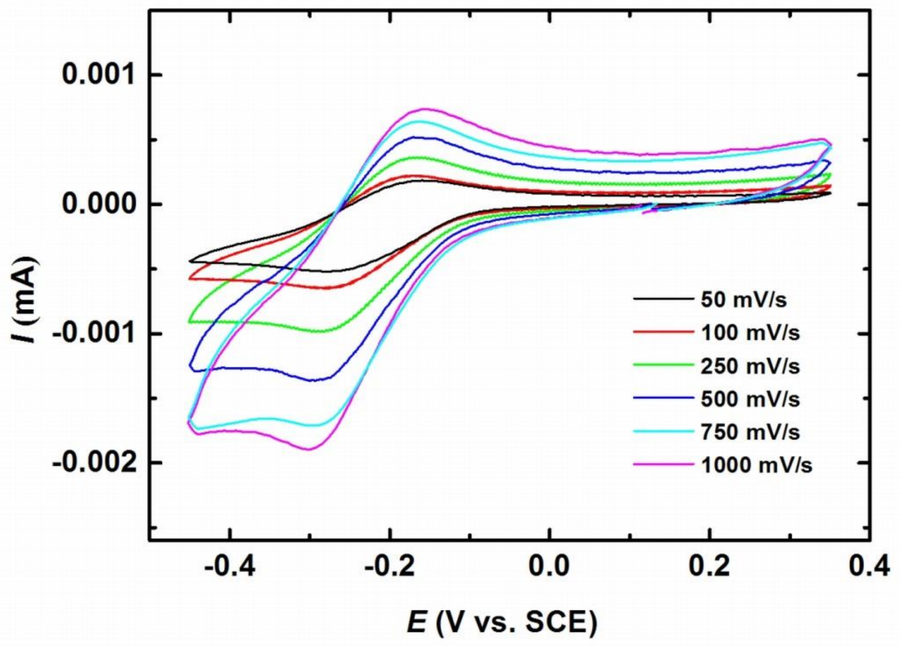

Figure S5: Cyclic voltammograms (cathodic scans) of 0.34 mM $Cu^{II}Br_2$/TPMA in 50% (v/v) *t*BA/DMF ([*t*BA] = 3.4 M) containing 0.2 M TBAP recorded at different scan rates (given next to the curves).

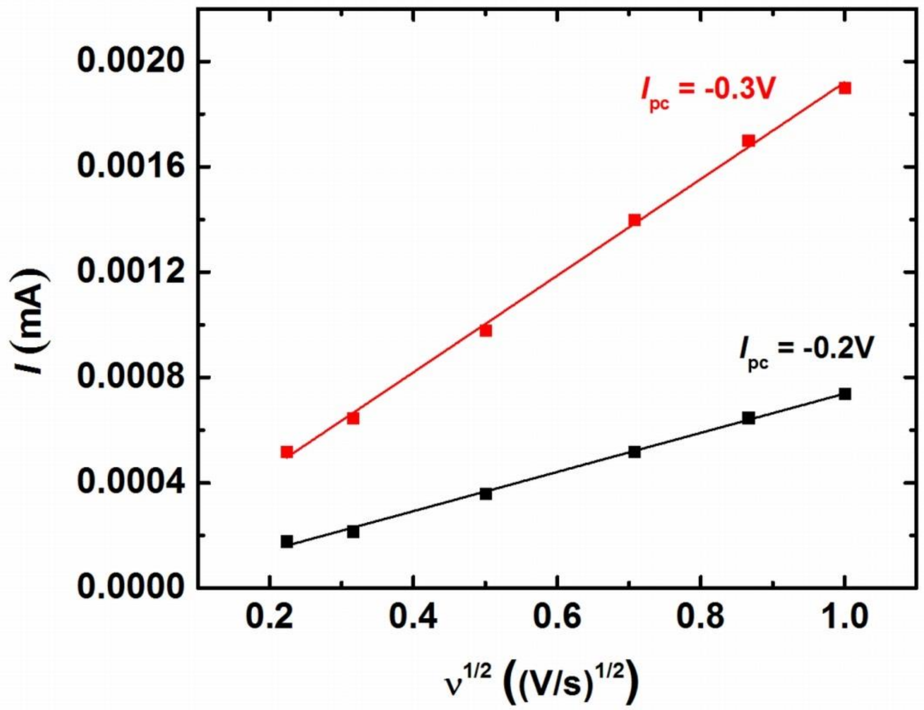

Figure S6: The dependence of the peaks current on square root of scan rate registered in the system: [*t*BA]/[$Cu^{II}Br_2$]/[TPMA] = 110/0.011/0.022, [*t*BA] = 3.4 M in DMF, [$Cu^{II}Br_2$/TPMA] = 0.34 mM, [TBAP] = 0.2 M for cathodic peak (–0.3 V) and reverse anodic peak (–0.2 V).

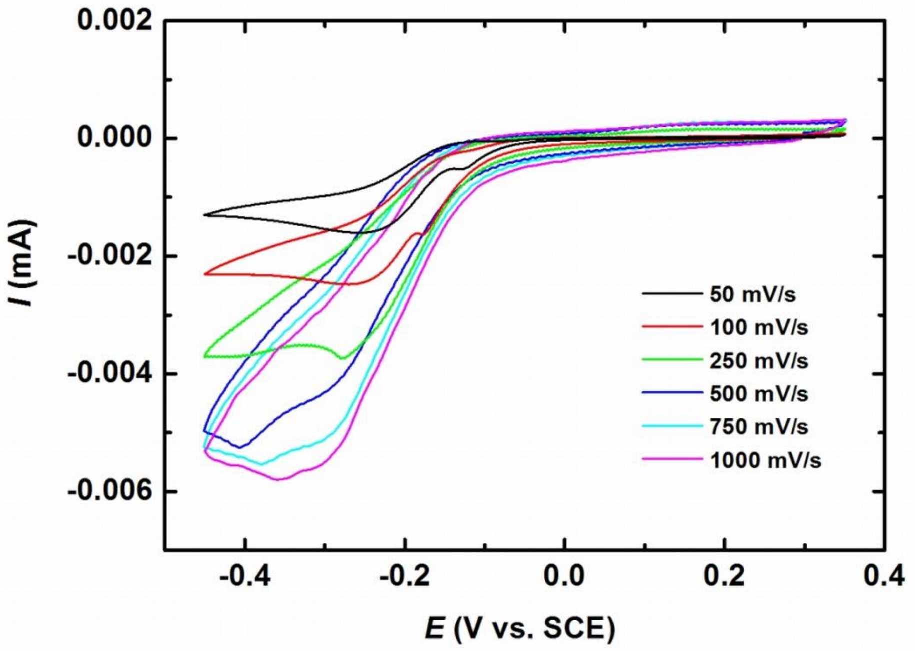

Figure S7: Cyclic voltammograms (cathodic scans) of 0.34 mM $Cu^{II}Br_2$/TPMA in 50% (v/v) *t*BA/DMF ([*t*BA] = 3.4 M) containing 0.2 M TBAP recorded at a different scan rates (given next to the curves) in the presence of 6.2 mM QC-Br_5.

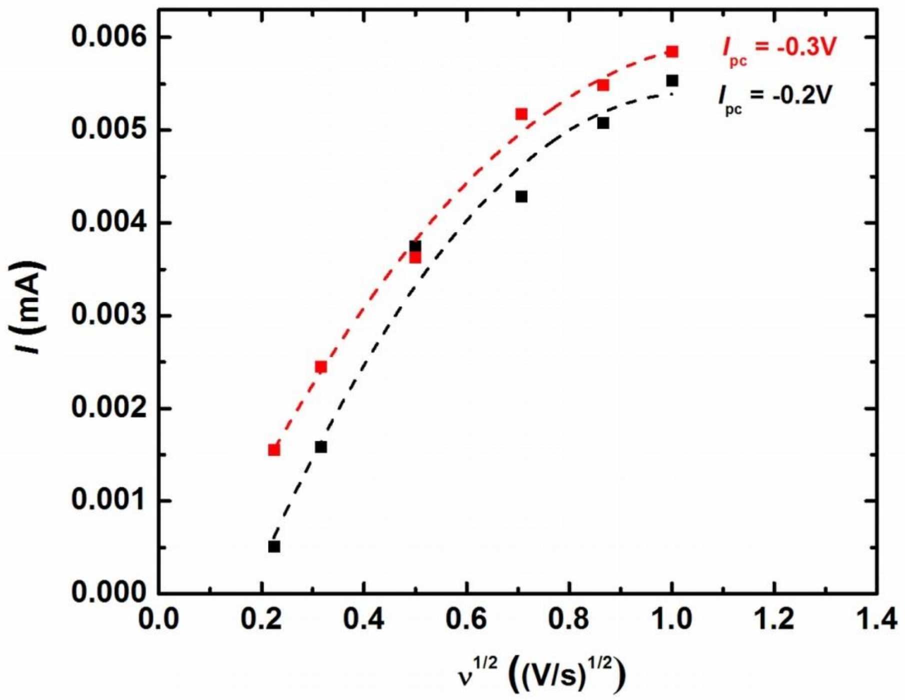

Figure S8: The dependence of the peaks current on square root of scan rate registered in the system: [*t*BA]/[QC-Br_5 (per 5 initiation sites)]/[$Cu^{II}Br_2$]/[TPMA] = 110/1/0.011/0.022, [*t*BA] = 3.4 M in DMF, [$Cu^{II}Br_2$/TPMA] = 0.34 mM, [TBAP] = 0.2 M for cathodic peak (–0.3 V) and reverse anodic peak (–0.2 V).

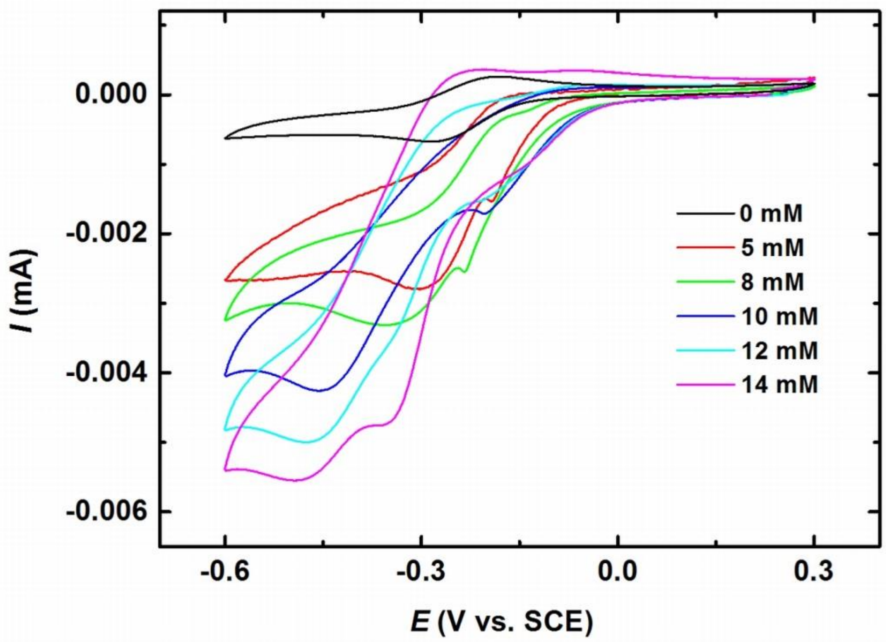

Figure S9: Cyclic voltammograms (cathodic scans) of 0.34 mM $Cu^{II}Br_2$/TPMA in 50% (v/v) *t*BA/DMF ([*t*BA] = 3.4 M) containing 0.2 M TBAP recorded at different scan rates (given next to the curves) in the presence of QC-Br_5. The concentration of QC is given next to the curves.

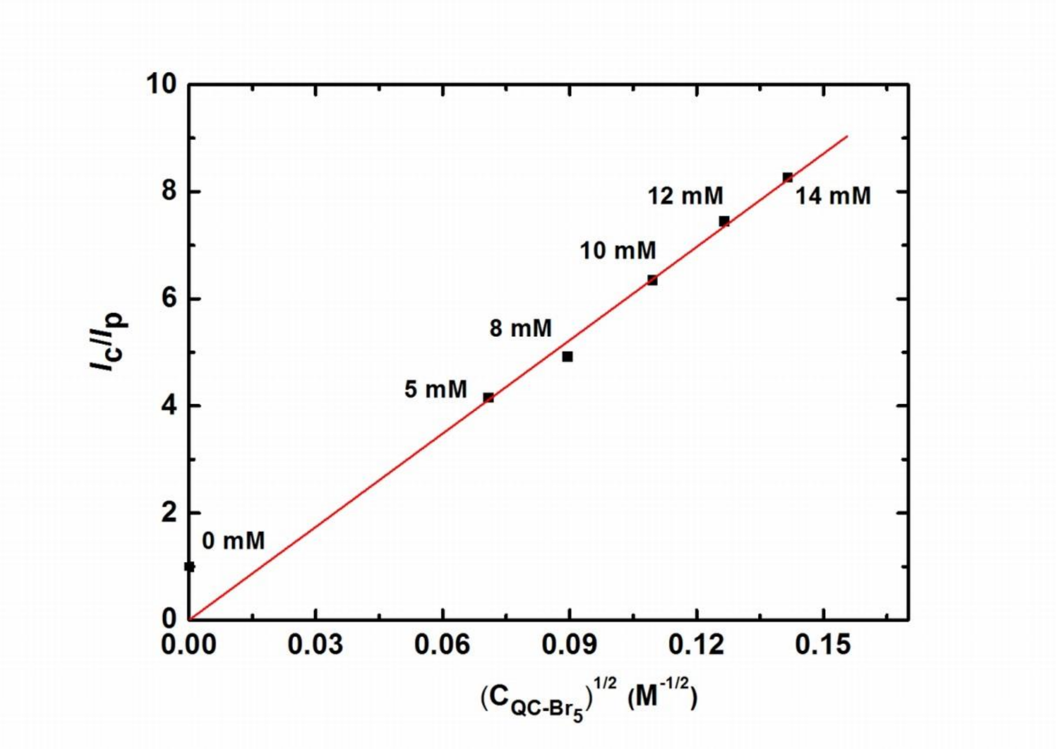

Figure S10: The dependence of the I_c/I_p ratio on square root of QC-Br_5 concentration [$M^{-1/2}$] in the system: [*t*BA]/[QC-Br_5 (per 5 initiation sites)]/[$Cu^{II}Br_2$]/[TPMA] = 110/1/0.011/0.022, [*t*BA] = 3.4 M in DMF, [$Cu^{II}Br_2$/TPMA] = 0.34 mM, [TBAP] = 0.2 M for cathodic peaks at –0.3 V. The concentrations of QC-Br_5 are given next to particular points.

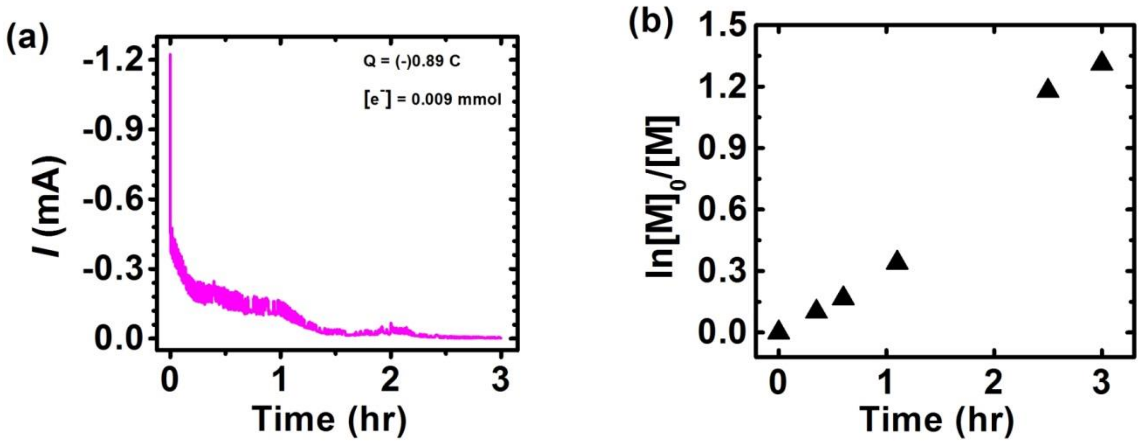

Figure S11: Synthesis of QC-(P*t*BA-Br)$_5$ polymers by *se*ATRP under constant potential conditions; (a) current profile vs. polymerization time, and (b) first-order kinetic plot of monomer conversion vs. time. Reaction conditions: [*t*BA]/[QC-Br$_5$ (per 5 initiation sites)]/[$Cu^{II}Br_2$]/[TPMA] = 110/1/0.011/0.022, [*t*BA] = 3.4 M, [$Cu^{II}Br_2$/TPMA] = 0.34 mM, [TBAP] = 0.2 M, T = 65°C. Table 1, entry 2.

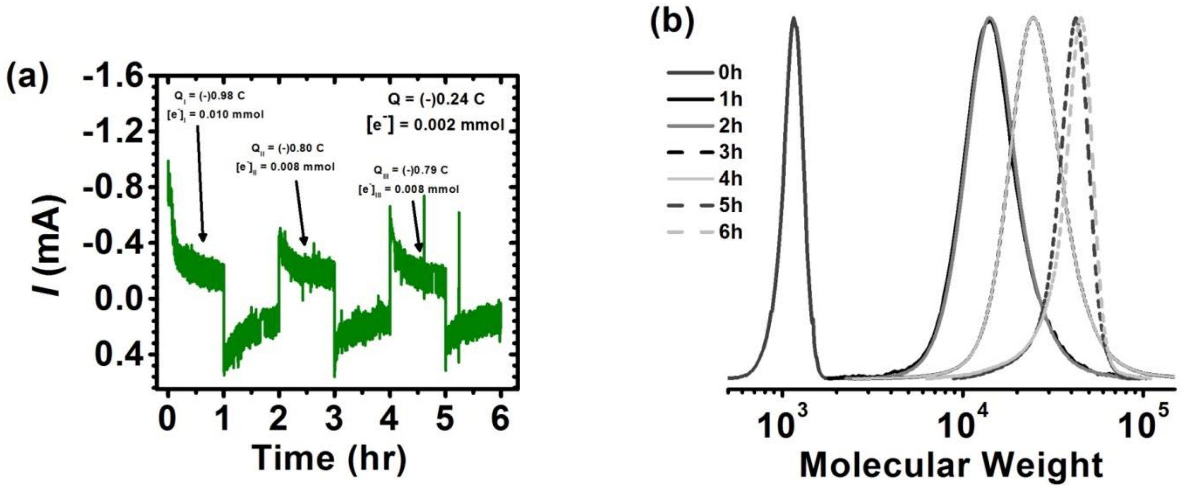

Figure S12: Synthesis of QC-(P*t*BA-Br)$_5$ polymers by *se*ATRP with periodically applied different values of potential, between –240 mV and 600 mV vs. SCE, respectively; (a) current profile vs. polymerization time, and (b) GPC traces of *t*BA polymerization and their evolution over time. Reaction conditions: [*t*BA]/[QC-Br$_5$ (per 5 initiation sites)]/[Cu^{II}Br$_2$]/[TPMA] = 110/1/0.011/0.022, [*t*BA] = 3.4 M, [Cu^{II}Br$_2$/TPMA] = 0.34 mM, [TBAP] = 0.2 M, T = 65°C. Table 1, entry 2.

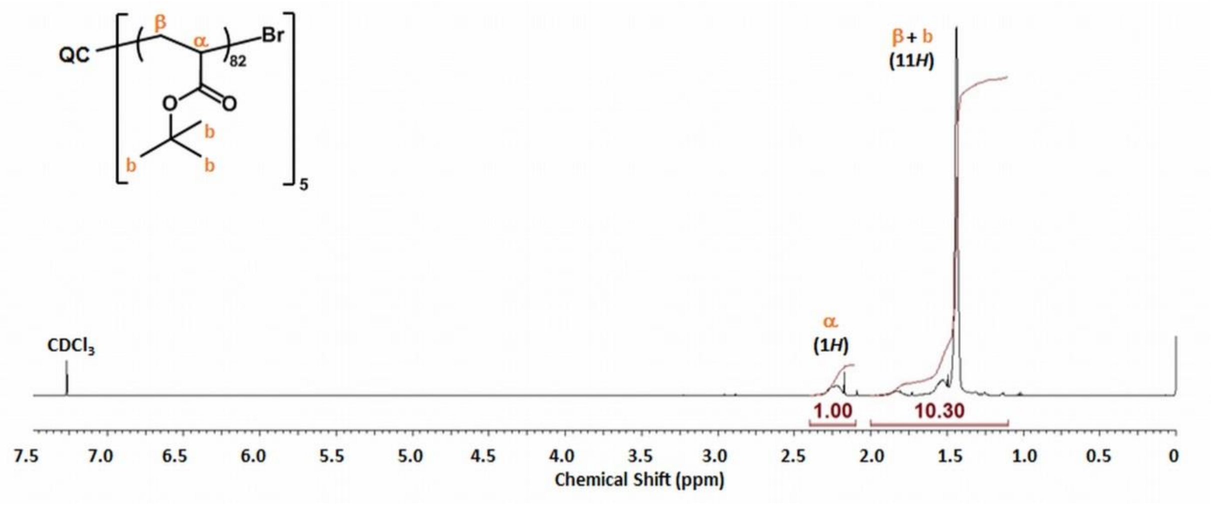

Figure S13: ^{1}H NMR spectrum of P*t*BA homopolymers grafted from quercetin-based macroinitiator (QC-(PBA_{82}-Br)$_5$; M_n = 53,600, *Đ* = 1.11) after purification (in $CDCl_3$). Table 1, entry 2.

References

1. Xia, J. H.; Matyjaszewski, K. *Macromolecules* **1999**, *32*, 2434-2437.
2. Park, S.; Chmielarz, P.; Gennaro, A.; Matyjaszewski, K. *Angew. Chem. Int. Ed.* **2015**, *54*, 2388-2392.
3. Leng, X.; Nguyen, N. H.; van Beusekom, B.; Wilson, D. A.; Percec, V. *Polym. Chem.* **2013**, *4*, 2995-3004.
4. Chmielarz, P.; Park, S.; Sobkowiak, A.; Matyjaszewski, K. *Polymer* **2016**, *88*, 36-42.
5. Chmielarz, P. *Chem. Pap.* **2017**, *71*, 161-170.
6. Chmielarz, P. *Express Polym. Lett.* **2017**, *11*, 140-151.
